# Meta‐analytic evidence for downregulation of the amygdala during working memory maintenance

**DOI:** 10.1002/hbm.25828

**Published:** 2022-03-29

**Authors:** Lycia D. de Voogd, Erno J. Hermans

**Affiliations:** ^1^ Donders Institute for Brain, Cognition, and Behavior Radboud University and Radboud University Medical Center Nijmegen The Netherlands

**Keywords:** amygdala, emotion regulation, functional MRI, meta‐analysis, working memory

## Abstract

The amygdala is a region critically implicated in affective processes. Downregulation of the amygdala is one of the hallmarks of successful emotion regulation. Top‐down inhibition of the amygdala is thought to involve activation of the executive control network. This reciprocal relationship, however, is not exclusive to explicit emotion regulation. It has been noted that any cognitively demanding task that activates executive control network may downregulate the amygdala, including a standard working memory task. Such downregulation is likely established in a load‐dependent fashion with more cognitive demand leading to stronger deactivation. Using a coordinate‐based meta‐analysis, we examined whether a standard working memory task downregulates the amygdala similarly to cognitive reappraisal. We found that a standard 2‐back working memory task indeed systematically downregulates the amygdala and that deactivated clusters strongly overlap with those observed during a cognitive reappraisal task. This finding may have consequences for the interpretation of the underlying mechanism of cognitive reappraisal: amygdala downregulation may be related to the cognitively demanding nature of reappraisal and not per se by the act of the reappraisal itself. Moreover, it raises the possibility of applying working memory tasks in clinical settings as an alternative emotion regulation strategy.

## Graphical abstract

Using a coordinate‐based meta‐analysis, we found that a standard 2‐back working memory task systematically downregulates the amygdala and that deactivated clusters strongly overlap with those observed during a cognitive reappraisal task. This finding may have consequences for the interpretation of the underlying mechanism of cognitive reappraisal: amygdala downregulation may be related to the cognitively demanding nature of reappraisal and not per se by the act of the reappraisal itself. Moreover, it raises the possibility of applying working memory tasks in clinical settings as an alternative emotion regulation strategy.

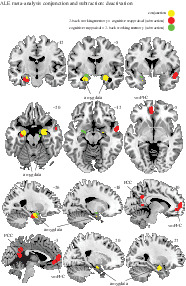

## 1 INTRODUCTION

Downregulation of the amygdala, a region critically implicated in threat detection (LeDoux, 1996; Öhman, 2005), is one of the hallmarks of successful emotion regulation. Cognitive regulation of emotion is accompanied by activation in the dorsolateral prefrontal cortex (dlPFC), a region that is part of the executive control network (Seeley et al., 2007), and by downregulation of the amygdala (Buhle et al., 2014). Since there are little or no direct connections between the dlPFC and the amygdala (Amaral, Price, Pitkanen, & Carmichael, 1992), it is commonly thought that downregulation may occur indirectly, for example, via the ventromedial prefrontal cortex (e.g., Diekhof, Geier, Falkai, & Gruber, 2011; Etkin, Büchel, & Gross, 2015; Phelps, Delgado, Nearing, & LeDoux, 2004; Schiller & Delgado, 2010), but see (Buhle et al., 2014).

However, this opposing interplay between the executive control network and the amygdala is not specific for emotion regulation. It has recently been noted that *any* cognitively demanding task that activates the executive control network may potentially downregulate the amygdala (de Voogd, Hermans, & Phelps, 2018). Indeed, a downregulation of the amygdala has been observed during the execution of a standard working memory task (de Voogd, Hermans, & Phelps, 2018; de Voogd et al., 2018), with more cognitive load leading to a stronger downregulation (Van Dillen, Heslenfeld, & Koole, 2009; de Voogd, Hermans, & Phelps, 2018).

Cognitively demanding tasks have also been shown to be accompanied by a downregulation of defensive responses to threat. When participants perform a standard *n*‐back working memory paradigm while simultaneously undergoing a threat conditioning paradigm, conditioned responses have been shown to be reduced (Carter, Hofstötter, Tsuchiya, & Koch, 2003). Moreover, threat‐potentiated startle responses are decreased when participants perform a working memory paradigm (King & Schaefer, 2011; Vytal, Cornwell, Arkin, & Grillon, 2012). Reductions in these threat‐potentiated startle responses are stronger when the cognitive demand is increased (Vytal et al., 2012). Finally, subjective ratings of negative mood after viewing aversive images (Van Dillen & Koole, 2007; Van Dillen et al., 2009) or subjective reports of state anxiety (Balderston et al., 2016; Vytal et al., 2012) also were shown to decrease with increasing cognitive load of a working memory task. These findings together suggest that cognitive demand, beyond mere attention reorientation or distraction, may play a role in the downregulation of the amygdala that is observed during emotion regulation.

Lesion studies in humans have indicated that such defensive responses to threat are (partly) dependent on the amygdala (Bechara et al., 1995; Klumpers, Morgan, Terburg, Stein, & van Honk, 2015; LaBar, LeDoux, Spencer, & Phelps, 1995). Therefore, a cognitively demanding task may offer a noninvasive way to impact defensive responses to threat via downregulation of the amygdala. Indeed, threat‐induced amygdala responses were shown to be attenuated during the execution of a cognitively demanding task (McRae, Chopra, Gabrieli, Gross, & Ochsner, 2010; Price, Paul, Schneider, & Siegle, 2013). Even though the general interpretation of such findings is that an initial amygdala activation, in response to the threat, can be downregulated by a cognitively demanding task, other findings show amygdala downregulation can also be observed without the presence of a threat‐induced amygdala response (de Voogd, Hermans, & Phelps, 2018; de Voogd, Kanen, et al., 2018). Thus, performing a working memory task alone is sufficient to downregulate the amygdala.

If a working memory task establishes a downregulation of the amygdala and defensive response to threat, it raises the question whether the effects of cognitive reappraisal on the amygdala are driven by cognitive demand. It has been proposed that through a reinterpretation of the threatening situation, with the explicit goal to change the affective impact of the threat, threat‐related responses and amygdala reactivity is reduced (Buhle et al., 2014). Alternatively, downregulation of the amygdala during cognitive reappraisal might be due to the cognitively demanding nature of the task and not per se by the act of the reappraisal itself (de Voogd, Hermans, & Phelps, 2018). This does not necessarily mean that if cognitive demand is indeed driving amygdala downregulation, it also is driving changes in self‐report. It is possible that changes in self‐report, apart from potential demand characteristics, may occur via other neural pathways also shown to be involved in regulating emotions (e.g., Etkin et al., 2015). Indeed, not all cognitive reappraisal studies report amygdala downregulation (Ochsner, Silvers, & Buhle, 2012). It remains unclear, however, whether downregulation of the amygdala is a consistent finding across studies on working memory. More importantly, it is unknown whether there is a systematic difference in amygdala downregulation between a working memory task and cognitive reappraisal.

The aim of this study is therefore to investigate, using a meta‐analytic approach, whether working memory tasks downregulate the amygdala, and whether this downregulation is similar to cognitive reappraisal. As a standard working memory task, we opted for a “2‐back” working memory task, as there are many studies available that have previously reported an activation (2‐back > control) contrast (Lee & Xue, 2018). To test whether a working memory task downregulates the amygdala similar to a cognitive reappraisal task, we conducted an activation likelihood estimation (ALE) coordinate‐based meta‐analysis (Eickhoff et al., 2009). We predicted a reduced blood oxygenation level‐dependent (BOLD) signal during a standard 2‐back working memory task that would overlap with the reduction in BOLD signal during cognitive reappraisal.

## 2 MATERIALS AND METHODS

### 2.1 Study and data selection for the ALE meta‐analysis

**FIGURE 1:**
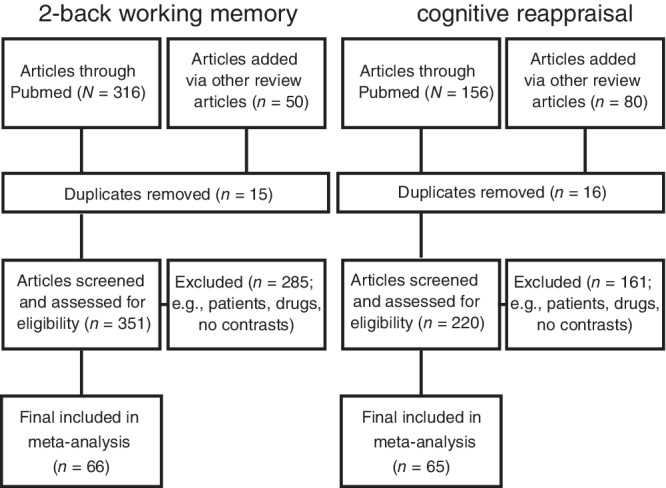
A flow chart describing the steps used to identify the articles that were included in the activation likelihood estimation (ALE) meta‐analysis

We performed the ALE meta‐analysis according to the Preferred Reporting Items for Systematic Reviews and Meta‐Analyses (PRISMA) guidelines (Moher et al., 2015). For the PRISMA flow diagram, see Figure 1.

#### 2.1.1 Eligibility criteria

Peer‐reviewed fMRI articles including healthy adult volunteers which included a 2‐back working memory or a cognitive reappraisal experiment.

#### 2.1.2 Information sources

The PubMed database (https://www.ncbi.nlm.nih.gov) and other meta‐analyses (Buhle et al., 2014; Kohn et al., 2014; Lee & Xue, 2018; Ochsner et al., 2012).

#### 2.1.3 Search

(a) ([2‐back (Title/Abstract)] AND fmri) NOT review (Publication Type), and (b) ([cognitive reappraisal (Title/Abstract)] AND fmri) NOT review (Publication Type). The search was performed on April 1, 2020.

#### 2.1.4 Study selection

Articles were included based on the following criteria: (a) healthy human adult volunteers (range between 18–45 mean years old). Articles including patient studies with a separate analysis of the control group were included, (b) whole‐brain analysis, (c) region of interest‐based analysis were excluded, except for the amygdala, (d) reporting of standardized coordinates for activation foci in Montreal Neurological Institute (MNI) or Talairach space, (e) working memory studies including a 2‐back condition: the specific modality is reported (see Table 1) OR emotion regulation strategy that involved cognitive reappraisal: the specific technique such as reinterpretation or distancing is reported (see Table 2) (f) general linear model (GLM) analysis involving a 2‐back < > control analysis: the control condition such as rest or 0‐back is reported   OR GLM analysis involving a Reappraisal < > control analysis: the specific instruction such as view, watch, or attend is reported.

**TABLE 1:** An overview of the working memory studies included in the meta‐analysis

Author	*N* participants (*N* females)	Source	Space	A/D	Age *M* (*SD*) or range	Domain	Stimuli	Activation contrast	Deactivation contrast
Allen et al. (2006)	10 (2F)	Table 2	Tal	A	23–35 range	Visual	Letters	Sham condition (2‐back > 0‐back)	
Barch et al. (2007)	120 (70F)	Table 4	Tal	A	27.2 (10.8)	Verbal, nonverbal	Words, faces	Working memory > encoding	
Binder et al. (2006)	12 (5F)	Table 2	Tal	A	23.52 (2.52)	Verbal	Letters	2‐back > 0‐back	
	12 (5F)	Table 3	Tal	A	23.52 (2.52)	Nonverbal	Abstract texture patterns	2‐back > 0‐back	
Bleich‐Cohen et al. (2014)	20 (8F)	Table 2a	Tal	A	26.4 (2.7)	Visual	Achromatic numbers	2‐back > 0‐back	
Blokland, et al. (2011)	319 (174F)	Table S1	MNI	A	23.6 (1.8)	Spatial	Numbers	2‐back > 0‐back	
Bustamante et al. (2011)	15 (0F)	Table 2	Tal	A	32.40 (7.56)	Auditory	Letters	2‐back > 0‐back	
Carlson et al. (1998)	7 (3F)	Table 1	Tal	A	21.1	Visuospatial	White squares	2‐back > 0‐back	
Chang et al. (2004)	10 (0F)	Table 2	Tal	A	14.4 (3.2)	Visuospatial	Letter O	2‐back > 0‐back	
Chang et al. (2010)	21 (0F)	Table 3	MNI	A	49.7 (4.3)	Visual	Letters (symbols from the Korean alphabet)	2‐back > rest	
Deckersbach et al. (2008)	17 (17F)	Table 3	MNI	A	25.6 (5.9)	Visual	Letters	*N*‐back > fixation (sad/neutral)	
Dehghan et al. (2019)	24 (12F)	Table 5	MNI	A/D	23 (2.69)	Visual	Letters	2‐back > 0‐back	0‐back > 2‐back
de Voogd et al. (2018)	24 (12F)	Table 1	MNI	A/D	26.95 (3.6)	Visual	Numbers	2‐back > fixation	Fixation > 2‐back
Dima et al. (2014)	40 (20F)	Table II	MNI	A	31.5 (10.4)	Visual	Letters	2‐back > 0‐back	
Dores et al. (2017)	10 (4F)	Table 2	Tal	A	27.10 (2.89)	Visuospatial	Grid	2‐back > fixation	
Drapier et al. (2008)	20 (10F)	Table 2	Tal	A	41.9 (11.6)	Visual	Letters	2‐back > 0‐back	
Drobyshevsky et al. (2006)	31 (15F)	Table 2	Tal	A	41 (15.3)	Visual	Letters	2‐back > 0‐back	
Fernandez‐Corcuera et al. (2013)	41 (17F)	Table 2	MNI	A/D	40.27 (9.8)	Visual	Letters	2‐back > baseline	Baseline > 2‐back
Ford et al. (2018)	32 (20F)	Table 3	MNI	A	30–65	Visual	Faces/places/tools/body parts	2‐back > 0‐back	
Garrett et al. (2011)	19 (6F)	Table 3	Tal	A	34.85 (12.54)	Visual	Letters	2‐back > 0‐back	
González‐Garrido et al. (2019)	18 (7F)	Table 4	MNI	A	21.11 (4.65)	Visual	Neutral faces	2‐back > rest	
	18 (7F)	Table 5	MNI	A	21.11 (4.65)	Visual	Happy faces	2‐back > rest	
	18 (7F)	Table 6	MNI	A	21.11 (4.65)	Visual	Fear faces	2‐back > rest	
Goikolea et al. (2019)	31 (15F)	Table 2	Tal	A/D	31.06 (8.76)	Visual	Letters	2‐back > baseline	Baseline > 2‐back
Guimond et al. (2018)	20 (5F)	Table 1S	MNI	A/D	25.05 (4.05)	Visual	Faces	2‐back > 0‐back	0‐back > 2‐back^a^
Habel et al. (2007)	22 (0F)	Table 4	MNI	A	30.77 (9.65)	Visual	Letters	2‐back > 0‐back	
Harding et al. (2016)	25 (11F)	Table 1	MNI	A	25.5 (4.4)	Visual	Numbers	2‐back > 0‐back	
Honey et al. (2000)	20 (0F)	Table 1	Tal	A	39.3 (13.6)	Visual	Letters	2‐back > 0‐back	
Honey et al. (2003)	27 (6F)	Table 3	Tal	A	35.1 (9.9)	Visual	Letters	2‐back > 0‐back	
Johannsen et al. (2013)	12 (8F)	Table 1	MNI	A	26.1 (4.7)	Visual	Letters	2‐back > 0‐back	
Joseph et al. (2012)	8 (8F)	Table 1	MNI	A	25 (6.4)	Visual	Letters	2‐back > 0‐back	
	8 (8F)	Table 1	MNI	A	25 (6.4)	Visual	Letters	2‐back > 0‐back	
Keresztes et al. (2004)	29 (20F)	Table 1	MNI	A	22.93 (2.26)	Visual	Letters	2‐back > 0‐back	
Kwon et al. (2001)	15 (15F)	Table 3	Tal	A	15.05 (4.58)	Visual	Letters	2‐back > 0‐back	
Koppelstaetter et al. (2008)	15 (0F)	Table 2	Tal	A	25–47 (5.58)	Visual	Letters	2‐back > 0‐back	
Li et al. (2014)	15 (15F)	Table S1	MNI	A	19.45 (1.38)	Visual	Letters	2‐back > rest	
Luo et al. (2014)	25 (0F)	Table I	MNI	A/D	23.14 (1.83)	Visual	Faces	2‐back > 0‐back	0‐back > 2‐back^a^
Lycke et al. (2008)	26 (14)	Table 1	Tal (from MNI)	A	23.4 (2.4)	Auditory	Letters	2‐back > rest	
	26 (14)	Table 1	Tal (from MNI)	A	23.4 (2.4)	Visuospatial	Letters	2‐back > rest	
Matsuo et al. (2007)	15 (10F)	Table 2	Tal	A	37.77 (12.1)	Visual	Numbers	2‐back > 0‐back	
Meisenzahl et al. (2006)	12 (1F)	Table 3	Tal	A	33.58 (9.27)	Visual	Letters	2‐back > 0‐back	
	12 (1F)	Table 3	Tal	A	33.58 (9.27)	Visual	Letters	2‐back > 0‐back (degraded)	
Monks et al. (2004)	12 (0F)	Table 1	Tal	A	45.6 (3.52)	Visual	Letters	2‐back > 0‐back	
Oflaz et al. (2014)	9 (2F)	Table 3	MNI	A	44.6 (10.2)	Visual	Letters	2‐back > 0‐back	
Park et al. (2011)	10 (0F)	Tables 4–6	MNI	A/D	23.7 (0.95)	Sound	Word	2‐back > 0‐back	0‐back > 2‐back
	10 (0F)	Tables 4–6	MNI	A/D	23.7 (0.95)	Sound	Pitch	2‐back > 0‐back	0‐back > 2‐back
	10 (0F)	Tables 4–6	MNI	A/D	23.7 (0.95)	Sound	Location	2‐back > 0‐back	0 back > 2‐back
Paskavitz et al. (2010)	17 (9F)	Table 1	Tal	A	35.08 (13.73)	Visual	Letters	2‐back > 0‐back	
Pfefferbaum et al. (2001)	10 (0F)	Table 2	Tal	A/D	60.2 (12.8)	Visual	Letters	2‐back > rest	Rest > 2‐back
Philip et al. (2016)	13 (9F)	Table 3B	Tal	A	30 (9)	Visual	Letters	2‐back > baseline	
Quidé et al. (2013)	28 (14F)	Table 2A	MNI	A	32.96 (10.97)	Visual	N/A	2‐back > 0‐back	
Ragland et al. (2002)	11 (5F)	Table 2	Tal	A	32.2	Visual	Letters	2‐back > 0‐back	
	11 (5F)	Table 2	Tal	A	32.2	Visual	Fractals	2‐back > 0‐back	
Rämä et al. (2001)	8 (8F)	Table 1	Tal	A	22	Auditory	Connotation	2‐back > 0‐back	
Reuter et al. (2008)	49 (30F)	Table 1	MNI	A	27.4 (6.3)	Visual	Numbers	2‐back > 0‐back	
Rodriguez‐Jimenez et al. (2009)	13 (6F)	Table 1	Tal (from MNI)	A	30 (8.19)	Auditory and visual conjunction	Letters	2‐back > 0‐back	
	13 (6F)	Table 1	Tal (from MNI)	A				2‐back > 0‐back	
Rudner et al. (2013)	20 (15F)	Table 5	Tal (from MNI)	A	26.4 (5.6)	Visual	Pictures	2‐back > baseline (phonological)	
	20 (15F)	Table 5	Tal (from MNI)	A	26.4 (5.6)	Visual	Pictures	2‐back > baseline (orthographic)	
Salavert et al. (2018)	41 (13F)	Table 3	MNI	A/D	31.7 (9.6)	Visual	Letters	2‐back > baseline	0‐back > 2‐back
Sanchez‐Carrion et al. (2008)	18 (7F)	Table 3	MNI	A	24.2 (4.7)	Visual	Numbers	2‐back > 0‐back	
Scheuerecker et al. (2008)	23 (4F)	Table 2	MNI	A	32.6 (9.9)	Visual	Letters	2‐back > 0‐back	
	23 (4F)	Table 2	MNI	A	32.6 (9.9)	Visual	Letters	2‐back degraded > 0‐back degraded	
Schneiders et al. (2011)	48 (26)	Table 1	Tal	A	23.67	Visual/auditory	Pattens/bird voice	2‐back > 0‐back pretest	
	48 (26)	Table 2	Tal	A	23.67	Visual/auditory	Pattens/bird voice	2‐back > 0‐back posttest	
Seo et al. (2012)	22 (22F)	Table 2	MNI	A/D	38.27 (8.48)	Visual	Letters	2‐back > 0‐back	0‐back > 2‐back
Seo et al. (2014)	34 (34F)	Table 2	MNI	A	59.3 (5.2)	Visual	Letters	2‐back > 0‐back	
Stoodley et al. (2012)	9 (0F)	Table 2	MNI	A	25.5	Visual	Letters	2‐back > 0‐back	
Stretton et al. (2012)	15 (11F)	Table 2	MNI	A/D	27 (19–58 range)	Visuospatial	Location of dots	2‐back > 0‐back	Progressive deactivation
Suchan et al. (2005)	13 (8F)	Table 2	Tal	A	26	Visual/auditory	Pictures	2‐back > 0‐back	
Sumowski et al. (2010)	18 (15F)	Table 1	Tal	A/D	43.8 (7)	Visual	Letters	2‐back > rest	Rest > 2‐back
Sweet et al. (2010)	12 (7F)	Table 1	Tal	A/D	38.67 (12.91)	Visual	Letters	2‐back > 0‐back	0‐back > 2‐back
Thermenos et al. (2011)	10 (5F)	Table 2	MNI	A/D	17.1 (1.4)	Visual	Letters	2‐back > 0‐back	0‐back > 2‐back
Thomas et al. (2005)	16 (1F)	Table 3	Tal	A	37.6 (6.3)	Visual	Letters	2‐back > 0‐back	
Townsend et al. (2010)	14 (8F)	Table 3	MNI	A	30.8 (6.0)	Visual	Letters	2‐back > 0‐back	
	14 (8F)	Table 3	MNI	A	30.8 (6.0)	Visual	Letters	2‐back > 0‐back	
Valera et al. (2005)	20 (8F)	Table 3	MNI	A	33 (10.6)	Visual	Letters	2‐back > 0‐back	
Wu et al. (2017)	45 (21F)	Table 2	MNI	A/D	24.07 (4.83)	Visual	Numbers	2‐back > 0‐back	0‐back > 2‐back
Yan et al. (2011)	28 (16F)	Table 1	Tal	A/D	20.4 (1.4)	Visuospatial	White squares	2‐back > 0‐back (SL group)	0‐back > 2‐back
	28 (16F)	Table 1	Tal	A/D	20.9 (1.5)	Visuospatial	White squares	2‐back > 0‐back (HL group)	0‐back > 2‐back
Ziemus et al. (2008)	9 (4F)	Table 2	Tal	A	44.2 (9.6)	Visual	Letters	2‐back > 0‐back	

*Note*: A and D represent activation and deactivation, respectively.

^a^ These studies contained emotional stimuli.

**TABLE 2:** An overview of the cognitive reappraisal studies included in the meta‐analysis

Author	*N* (females)	Source	Space	A/D	Age *M* (*SD*)	Stimuli	Instruction	Activation contrast	Deactivation contrast
Albein‐Urios et al. (2013)	21 (1F)	Table S2	MNI	A	31.00 (4.60)	Negative and neutral pictures	Reappraise	Suppress > maintain	
Allard et al. (2014)	34 (16F)	Table 2	Tal	A/D	23.40 (4.39)	Unpleasant film clips	Reappraise	Emotion regulation > passive viewing	Passive viewing > emotion regulation
Beauregard et al. (2001)	10 (0F)	Table 2	Tal	A	23.5	Erotic movies	Decrease/distance	Attempted inhibition condition > neutral	
Campbell‐Sills et al. (2011)	26 (22F)	Table 1	Tal	A	19.15 (1.83)	Negative pictures	Reappraise	Reduce > maintain	
Che (2015)	29 (15F)	Table 1	MNI	A	22.62 (1.59)	Negative pictures	Reappraise/decrease	Reduce > maintain	
Corbalan et al. (2015)	17 (9F)	Table 3/text	MNI	A/D	41.4 (13.3)	Negative and neutral pictures	Reappraise	Decrease > look	Look > decrease
Cosme et al. (2018)	33 (16F)	Table 3	MNI	A/D	18.12 (0.34)	Food pictures	Reappraise	Regulate > look	Look > regulate
de Wit et al. (2015)	38 (20F)	Table 2	MNI	A	39.6 (11.4)	Fear, OCD‐related, neutral pictures	Reappraise	Attend > regulate	
Delgado et al. (2008)	12 (6F)	Table 2	Tal	A/D	23.29 (3.31)	Conditioned stimulus with shock	Reappraise	Regulate CS+ > attend CS+	Attend > regulate CS+ trials
Denny et al. (2015)	21 (11F)	Table S1	MNI	A	29 (6.71)	Negative and neutral pictures	Reappraise	Reappraise cue > look cue	
	21 (11F)	Table S3	MNI	A	29 (6.71)	Negative and neutral pictures	Reappraise	Reappraise negative > look negative	
Domes et al. (2010)	33 (17F)	Table IV	MNI	A	m: 25.2 (1.9); f: 24.6 (1.6)	Negative pictures	Reappraise	Decrease > maintain	
Eippert et al. (2007)	24 (24F)	Tables II and III	MNI	A/D	23.3	Negative pictures	Reappraise/distance	Decrease > view	
Erk et al. (2010)	17 (8F)	Table 2	MNI	A/D	43.9 (10.1)	Negative and neutral pictures	Reappraise	Regulation > no regulation	Negative no regulation > regulation
	17 (8F)	Table 2	MNI	A/D	43.9 (10.1)	Negative and neutral pictures	Reappraise		Negative no regulation > regulation
Fitzgerald et al. (2018)	49 (67%F)	Table 2	MNI	A	25.24 (7.98)	Negative and neutral pictures	Reappraise	Reappraise > look‐negative	
Giuliani et al. (2014)	55 (33F)	Table 1	MNI	A/D	22.17 (2.36)	Food pictures	Reappraise	Regulate > look	Look > regulate
Goldin et al. (2008)	17 (17F)	Table 2	Tal	A	22.7 (3.5)	Negative film clips	Reappraise	Reappraise > watch‐negative (early)	
Goldin et al. (2019)	35 (20F)	Table 3	Tal	A/D	32.2 (8.9)	Autobiographical social situations	Reappraise	Reappraisal > react	React > reappraisal
Golkar et al. (2012)	58 (32F)	Table S1	MNI	A	24.02 (2.26)	Negative and neutral pictures	Reappraise	Reappraise > attend	
Grecucci et al. (2012)	21 (10F)	Table 2	MNI	A	23.5 (3.6)	Ultimate game/unfair offers	Reappraise	Unfair accepted down > look	
Hallam et al. (2015)	20	Table 3	Tal	A/D	20 (?)	Negative and neutral pictures	Reappraise	Implementation intention > goal intention	Goal intention > implementation intention
Harenski and Hamman (2006)	10 (10F)	Table 3	MNI	A	18–29	Moral and nonmoral pictures	Reappraise	Decrease moral > odd‐even baseline	
	10 (10F)	Table 3	MNI	A	18–29	Moral and nonmoral pictures	Reappraise	Decrease nonmoral > odd‐even baseline	
Hayes et al. (2010)	25 (11F)	Tables 1 and 2	MNI	A/D	21.6 (2.5)	Negative pictures	Reappraise	Reappraise > view	View > reappraise
Hollmann et al. (2012)	17 (17F)	Table 1	MNI	A	25.3 (3.1)	High‐caloric food pictures	Reappraise	Regulate tasty > admit tasty	
Kanske et al. (2011)	30 (17F)	Table 3	MNI	A/D	21.8 (2.1)	Negative and positive pictures	Reappraise	Reappraisal > view emotional	View emotional > reappraisal
Kanske et al. (2012)	25 (18F)	ST2	MNI	A/D	43.88 (11.21)	Negative and positive pictures	Reappraise	Reappraisal positive > view positive	View positive > reappraisal positive
	25 (18F)	ST2	MNI	A/D	43.88 (11.21)	Negative and positive pictures	Reappraise	Reappraisal negative > view negative	View negative > reappraisal negative
Kim and Hamann (2007)	10 (10F)	Table 3	MNI	A	20.7	Negative and positive pictures	Reappraise	Decrease > watch contrast for negative pictures	
	10 (10F)	Table 4	MNI	A	20.7	Negative and positive pictures	Reappraise	Decrease > watch contrast for positive pictures	
Koenigsberg et al. (2010)	16 (9F)	Table 1	MNI	A/D	31.8 (7.7)	Negative and neutral pictures	Reappraise/distance	Distancing ≥ looking	Looking ≥ distancing
Korb et al. (2015)	18 (10F)	Table 3	MNI	A/D	27	Angry prosody	Reappraise	Decrease > feel negative	Feel negative > decrease
Krendl et al. (2012)	20 (10F)	Table 1	MNI	A/D	21.6	(Non)stigmatized negative pictures	Reappraise	Decrease IAPS > attend IAPS	Attend IAPS > decrease IAPS
Lang et al. (2012)	15 (15F)	Table S3	MNI	A	24.73 (5.64)	Negative and neutral scripts	Reappraise/distance	Down > maintain	
Leiberg et al. (2012)	24 (24F)	Table S2	MNI	A/D	24.1	Negative and neutral pictures	Reappraise/distance	Disengage > view	View > disengage
Mak et al. (2009)	12 (12F)	Table 1	MNI	A/D	24 (1.78)	Positive pictures	Reappraise	Regulate > view	View > regulate
	12 (12F)	Table 1	MNI	A/D	24 (1.78)	Negative pictures	Reappraise	Regulate > view	View > regulate
McRae et al. (2010)	18	Table 3	MNI	A	24.4 (3.5)	Negative and neutral pictures	Reappraise	Reappraise > look	
McRae et al. (2008)	25 (13F)	Table 1	MNI	A	m: 20.36 and f: 20.6	Negative pictures	Reappraise	Decrease negative > look negative	
Modinos et al. (2010)	18 (7F)	Table 1	MNI	A	21.1 (2.8)	Negative and neutral pictures	Reappraise	Reappraisal > negative	
Moodie et al. (2020)	30 (17F)	Table 2	MNI	A	24.3	Negative and neutral pictures	Reappraise	Reappraisal > watch (low)	Watch > reappraisal (low)
	30 (17F)	Table 2	MNI	A	24.3	Negative and neutral pictures	Reappraise	Reappraisal > watch (high)	Watch > reappraisal (high)
Morawetz et al. (2017)	23 (12F)	Table 2	Tal	A/D	25.70 (5.95)	Negative and neutral pictures	Reappraise/distance	Decrease > look negative	Look negative > decrease
Nelson et al. (2015)	22 (11F)	Table 1	MNI	A	25.2 (5.8)	Negative and neutral pictures	Reappraise	Reappraise > maintain	
New et al. (2009)	14 (14F)	Table S3	MNI	A	31.7 (10.3)	Negative pictures	Reappraise	Diminish > maintain	
Ochsner et al. (2002)	15 (15F)	Tables 1 and 2	MNI	A/D	21.9	Negative and neutral pictures	Reappraise	Reappraise > attend	Attend > reappraise
Ochsner et al. (2004)	24 (24F)	Tables 2 and 3	MNI	A/D	20.6	Negative pictures	Reappraise	Decrease > look	Look > decrease
Otto et al. (2014)	26 (26F)	Table 1	Tal	A/D	24.9 (5.6)	Fearful faces + emotional information	Reappraise	Reappraise > look	Look > reappraise
Paret et al. (2011)	21 (0F)	Table 1	MNI	A	28 (4)	Shock or no shock	Reappraise	Main effect of reappraisal (R–NR)	
Paschke et al. (2016)	108 (55F)	Table S5 + text	MNI	A/D	26.12 (3.7)	Negative and neutral pictures	Distance	RegulateNeg > watchNeg	WatchNeg > regulateNeg
Phan et al. (2005)	14 (8)	Table 1	MNI	A/D	27.6 (4.4)	Negative pictures	Reappraise	S > M	M > S
Price et al. (2013)	11 (8F)	Table 2	Tal	A/D	22.2 (2.2)	Autobiographical memories	Reappraise	Reappraisal > fixation	Fixation > reappraisal
Qu et al. (2017)	29 (14F)	Table 1	MNI	A	19.2	Negative pictures	Reappraise	Decrease‐look (positive activation)	
	29 (14F)	Table 1	MNI	A	19.2	Negative pictures	Reappraise	Decrease‐look (negative activation)	
Sarkheil et al. (2015)	14 (8F)	Table 2	Tal	A/D	Range 20–27	Negative pictures	Reappraise	Reappraisal > view	View > reappraisal
Schardt et al. (2010)	37 (37F)	Table 1	MNI	A/D	22.6 (2.2)	Fear, disgust, neural pictures	Reappraise	Regulation > perception	Perception > regulation
Schienle et al. (2017)	45F	Table 1	MNI	A	22.91 (3.21)	Disgusting and neutral pictures	Reappraise	Reappraisal > passive viewing	
Schulze et al. (2011)	15F	Table S2	MNI	A/D	24.53 (2.85)	Negative and neutral pictures	Reappraise	Decrease > maintain emotions HC	Maintain > decrease emotions HC
Shermohammed et al. (2017)	25 (12F)	Table 3	MNI	A	20.89 (1.71)	Negative pictures	Reappraise	Decrease‐negative > look‐negative	
Silvers et al. (2015)	30 (13F)	Table 1	MNI	A	21.97	Negative and neutral pictures	Reappraise	Reappraise/low > look/low	
	30 (13F)	Table 1	MNI	A	21.97	Negative and neutral pictures	Reappraise	Reappraise/high > look/high	
Simsek et al. (2017)	15	Table 3	MNI	A	22.53 (1.80)	Negative and neutral pictures	Reappraise	Reappraise Neg > attend negative	
Sokol‐Hessner et al. (2013)	16	Table 1	Tal	A	19.8 (3.1)	Monetary decisions	Reappraise	Regulate decision ME > attend decision ME	
	14	Table S3	Tal	A/D	19.8 (3.1)	Monetary decisions	Reappraise	Regulate lose ME > attend lose ME	Attend lose ME > regulate lose ME
Sripada et al. (2014)	49 (23F)	Table 3	MNI	A/D	23.63 (1.3)	Aversive or neutral pictures	Reappraise	Reappraise > maintain	Maintain > reappraise
Staudinger et al. (2009)	16 (8F)	In text	MNI	A	23.1 (3.1)	Reward anticipation	Reappraise/distance	Distance > permit	
Staudinger et al. (2011)	24 (13F)	Table 1	MNI	A	25.1 (2.8)	Reward anticipation	Reappraise/distance	Regulate > permit	
Van der Meer et al. (2014)	20 (6F)	Table 3	MNI	A	35.5 (11.7)	Negative and neutral pictures	Reappraise	Reappraise > attend negative HC	
Van der Velde et al. (2015)	51	Table S1	MNI	A	37.1 (10.3)	Negative and neutral pictures	Reappraise	Reappraise > attend negative	
Van der Velde et al. (2015)	16 (8F)	Table S1	MNI	A	22.1 (3.6)	Negative and neutral pictures	Reappraise	Reappraise > attend negative HC	
Vanderhasselt et al. (2013)	42 (42F)	Table 1	MNI	A	21.26 (2.29)	Negative pictures	Reappraise	Target reappraisal > target appraise	
Walter et al. (2009)	18 (18F)	Table 1	MNI	A/D	24 (3)	Negative and neutral pictures	Reappraise	Regulation > no regulation	No regulation > regulation
Winecoff et al. (2013)	31 (21F)	Table 1	MNI	A	25	Negative and positive pictures	Reappraise	Negative regulate > negative experience (exp1)	
	31 (21F)	Table 1	MNI	A/D	25	Negative and positive pictures	Reappraise	Positive regulate > positive experience (exp1)	Positive experience > positive regulate
Ziv et al. (2013)	27 (13F)	Table 2	Tal	A	32.6 (9.5)	Pictures of faces	Reappraise	HC only: Reappraise > react (faces task)	
	27 (13F)	Table 3	Tal	A	32.6 (9.5)	Pictures of faces	Reappraise	HC only: Reappraise > react (criticism task)	

*Note*: A and D represent activation and deactivation, respectively.

**TABLE 3:** Significant clusters from the ALE meta‐analysis showing an activation pattern

Cluster #	Region	Side	*X* (mm)	*Y* (mm)	*Z* (mm)	mm^3^	ALE	*p*	Peak *Z*
WM activation (2‐back > control)									
#1	Anterior insula/dlPFC	L	−32	22	0	23,680	0.078	4.95E‐21	9.34
			−42	8	30		0.076	3.56E‐20	9.13
			−40	−8	40		0.046	6.95E‐11	6.42
			−28	−2	52		0.043	5.97E‐10	6.08
			−30	−8	48		0.039	7.72E‐09	5.66
			−36	38	24		0.034	1.90E‐07	5.08
#2	Anterior insula/dlPFC	R	30	6	58	18,840	0.069	6.53E‐18	8.54
			40	28	30		0.062	1.11E‐15	7.93
			30	−2	48		0.057	6.43E‐14	7.41
			32	38	22		0.042	1.63E‐09	5.92
			32	46	20		0.039	1.08E‐08	5.60
			36	6	32		0.039	1.09E‐08	5.60
			44	12	26		0.029	2.67E‐06	4.55
			22	−12	58		0.020	4.90E‐04	3.30
#3	Posterior parietal cortex/angular gyrus	L	−42	−44	42	12,440	0.090	2.69E‐25	10.33
			−28	−60	38		0.077	1.38E‐20	9.23
			−34	−54	46		0.070	2.27E‐18	8.66
			−20	−70	54		0.021	2.58E‐04	3.47
#4	Posterior parietal cortex/angular gyrus	R	30	−62	44	12,296	0.073	2.36E‐19	8.92
			40	−46	42		0.071	1.07E‐18	8.75
#5	Dorsal anterior cingulate cortex	L/R	−2	8	50	9,032	0.063	5.03E‐16	8.03
			8	26	32		0.029	3.54E‐06	4.49
#6	Anterior insula	R	32	22	−2	5,104	0.123	4.82E‐38	12.84
#7	Cerebellum		30	−62	−32	3,688	0.036	6.64E‐08	5.28
			26	−60	−20		0.030	1.49E‐06	4.67
			40	−62	−18		0.027	9.14E‐06	4.28
#8	Fusiform gyrus	L	−40	−60	−18	2,240	0.032	7.37E‐07	4.81
			−32	−64	−30		0.027	1.07E‐05	4.25
#9	Caudate/putamen	L	−16	−2	16	1,464	0.034	1.53E‐07	5.12
#10	Middle frontal gyrus	L	−36	56	14	1,456	0.037	3.24E‐08	5.41
CR activation (reappraisal > control)									
#1	Dorsal anterior cingulate cortex	L/R	−6	14	62	10,880	0.072	6.66E‐19	8.80
			12	18	62		0.037	2.28E‐08	5.47
			4	28	40		0.031	7.13E‐07	4.82
			20	12	60		0.029	2.46E‐06	4.57
			−6	24	44		0.024	2.70E‐05	4.04
			−2	36	38		0.024	3.30E‐05	3.99
			2	20	46		0.022	1.06E‐04	3.70
#2	Anterior insula	L	−46	28	−8	9,136	0.063	2.81E‐16	8.10
			−52	22	−2		0.045	1.06E‐10	6.35
			−42	46	−6		0.037	1.92E‐08	5.50
#3	dlPFC	L	−44	6	48	7,320	0.051	2.46E‐12	6.91
			−40	20	46		0.039	5.25E‐09	5.72
#4	Anterior insula	R	50	30	−8	6,552	0.059	7.60E‐15	7.69
			48	44	−10		0.035	4.38E‐08	5.35
			50	18	−4		0.034	9.59E‐08	5.21
			58	24	6		0.028	3.94E‐06	4.47
			40	22	−12		0.021	1.59E‐04	3.60
#5	Middle temporal gyrus/angular gyrus	L	−42	−56	22	5,488	0.039	5.08E‐09	5.73
			−56	−52	44		0.036	4.20E‐08	5.36
			−50	−64	42		0.034	8.50E‐08	5.23
			−52	−62	34		0.032	3.04E‐07	4.99
			−60	−52	20		0.023	7.03E‐05	3.81
			−62	−50	32		0.023	7.37E‐05	3.80
#6	Middle temporal gyrus	L	−60	−38	−4	4,768	0.063	5.24E‐16	8.02
#7	Angular gyrus	R	60	−54	38	3,768	0.051	1.90E‐12	6.94
#8	dlPFC	R	40	22	44	2,712	0.038	1.01E‐08	5.61
			50	6	46		0.027	7.55E‐06	4.33
			44	12	44		0.024	2.76E‐05	4.03
#9	Middle cingulate cortex	L/R	−2	−22	28	1,008	0.035	5.94E‐08	5.30

*Note*: All coordinates are defined in MNI152 space. All statistics listed are significant at *p* < .05, whole‐brain FWE‐corrected using a cluster‐forming threshold of *p* < .0001 uncorrected, and a permutation test with 1,000 permutations.

Abbreviations: ALE, activation likelihood estimation; dlPFC, dorsolateral prefrontal cortex; FWE, family‐wise error.

**TABLE 4:** Significant clusters from the ALE meta‐analysis showing a deactivation pattern

Cluster #	Region	Side	*X* (mm)	*Y* (mm)	*Z* (mm)	mm^3^	ALE	*p*	Peak *Z*
WM deactivation (control > 2‐back)									
#1	Posterior cingulate cortex/precuneus	L/R	−4	−50	30	5,568	0.031	1.68E‐11	6.63
			−4	−52	12		0.019	6.18E‐07	4.85
			4	−50	18		0.013	5.53E‐05	3.87
			−6	−60	16		0.011	2.55E‐04	3.48
			16	−56	30		0.011	3.67E‐04	3.38
			8	−58	20		0.010	6.24E‐04	3.23
#2	Ventromedial prefrontal cortex (vmPFC)	L/R	−6	58	10	5,480	0.028	3.27E‐10	6.18
			−6	46	−4		0.020	3.88E‐07	4.94
			4	62	14		0.016	4.97E‐06	4.42
			−2	52	−16		0.010	6.57E‐04	3.21
#3	Amygdala/hippocampus	L	−24	−8	−22	1,952	0.023	1.56E‐08	5.53
#4	Amygdala	R	24	−6	−20	1,160	0.024	6.16E‐09	5.70
#5	Angular gyrus	L	−48	−64	28	1,120	0.022	3.73E‐08	5.38
#6	Middle/superior temporal gyrus	R	54	4	−16	872	0.016	1.03E‐05	4.26
			58	4	−12		0.015	1.88E‐05	4.12
CR deactivation (control > reappraisal)									
#1	Amygdala/dorsal entorhinal cortex (BA34)	R	26	−4	−20	3,960	0.045	6.57E‐15	7.70
			18	−8	−16		0.028	9.83E‐09	5.62
#2	Amygdala/dorsal entorhinal cortex (BA34)	L	−24	−6	−18	3,000	0.058	1.78E‐20	9.20
#3	Thalamus/parahippocampal gyrus	L	−22	−28	−4	688	0.026	3.77E‐08	5.38

*Note*: All coordinates are defined in MNI152 space. All statistics listed are significant at *p* < .05, whole‐brain FWE‐corrected using a cluster‐forming threshold of *p* < .0001 uncorrected, and a permutation test with 1,000 permutations.

Abbreviations: ALE, activation likelihood estimation; FWE, family‐wise error.

#### 2.1.5 Data collection process

We performed an analysis on 66 working memory studies and 65 emotion regulation studies (see Tables 1 and 2). All studies reported an activation contrast (2‐back: 954 foci, 80 experiments, and 1,979 participants; cognitive reappraisal: 799 foci, 76 experiments, and 1,892 participants), but 16 (165 foci, 19 experiments, and 424 participants) 2‐back working memory studies and 29 (289 foci, 34 experiments, and 906 participants) emotion regulation studies reported a deactivation contrast. Two 2‐back studies included emotional faces as stimuli (see Table 1 indicated with superscript letter a). Since these can be considered as potentially threatening stimuli, we reran the analysis without these two studies to ensure our findings were not driven by these two studies. The results and conclusions remained the same and we therefore included those studies in the final analysis. None of the studies reported a deactivation contrast without an activation contrast.

#### 2.1.6 Data items

We collected the peak coordinates of the selected contrasts for analysis. The focus of this study are the deactivation contrasts (control > 2‐back and control > reappraise). We also included the activation contrasts, mainly for comparison purposes to several other meta‐analyses as a validation of our procedure. See Tables 1 and 2, for the articles included in the ALE meta‐analysis.

### 2.2 The ALE meta‐analysis procedure

We performed the meta‐analysis using the ALE algorithm implemented in the software GingerALE version 3.0.2 (Eickhoff, Bzdok, Laird, Kurth, & Fox, 2012; Eickhoff et al., 2009; http://www.brainmap.org/ale; Turkeltaub et al., 2012). ALE is a coordinate‐based method used for performing meta‐analyses of human brain imaging studies. A full‐width half‐maximum of the Gaussian function is used to blur the foci. The size of the gaussian is determined by the number of subjects in each experiment. An ALE image is created based on all coordinates. Significance is determined via a permutation procedure which we set to 1,000 permutations. We used a cluster‐forming voxel‐level threshold of *p* < .001 (uncorrected). Alpha was set at .05, whole‐brain family‐wise error corrected at the cluster level. Before the analysis, we converted all coordinates in Talairach space to MNI space using the GingerALE foci converter tool. The analyses were done on the MNI coordinates.

In addition, we performed a comparison analysis on the deactivation contrasts (control > 2‐back and control > reappraise) including a conjunction and subtraction analysis. In the conjunction analysis, a conjunction image was created using the voxel‐wise minimum value of the two contrast (control > 2‐back and control > reappraise) ALE maps. The conjunction output image shows the similarity in clusters between the two contrast maps. In the subtraction analysis, two contrast (control > 2‐back and control > reappraise) ALE maps are directly subtracted from each other. In addition, we performed a “pooled” analysis following the procedure described above, including the coordinates from both contrasts. The pooled data were subsequently used for permutation testing where the data were randomly assigned to one of the two contrasts and repeated 10,000 times, false discovery rate  < 0.05, minimum volume = 0 mm^3^. The subtraction maps were tested against this null distribution.

Lastly, as a control analysis, we investigated whether the instruction to increase one's emotion would, similar to decreasing one's emotion (as described above), affect activation in the executive control network and amygdala. In total, 10 studies of the 65 cognitive reappraisal studies also included a condition in which participants were required to increase their emotions. We performed the ALE meta‐analysis as described above on two contrasts, namely the reappraise increase > control (177 foci, 11 experiments, and 209 subjects) and the control > reappraise increase (6 foci, 4 experiments, and 86 subjects).

Anatomical labels provided by the GingerALE software are derived from the Talairach Daemon atlas (talairach.org). For the amygdala deactivation clusters, we reported the percentage of that cluster falling in the amygdala based on those labels.

## 3 RESULTS

### 3.1 ALE meta‐analysis activation contrasts

We found 10 clusters for the 2‐back > control contrast among which are located in the left (cluster #1, *z* = 9.34, *p* = 4.95E‐21, and mm^3^ = 23,680) and right (cluster #2, *z* = 8.44, *p* = 6.53E‐18, and mm^3^ = 18,840) dlPFC; the left (cluster #3, *z* = 10.32, *p* = 2.69E‐25, and mm^3^ = 12,440) and right (cluster #4, *z* = 9.92, *p* = 2.36E‐19, and mm^3^ = 12,296) posterior parietal cortex; the left (cluster #1, *z* = 9.34, *p* = 4.95E‐21, and mm^3^ = 23,680) and right (cluster #2, *z* = 8.44, *p* = 6.53E‐18, and mm^3^ = 18,840 and cluster #6, *z* = 12.84, *p* = 4.82E‐38, and mm^3^ = 5,104) anterior insula; and the left/right (cluster #5, *z* = 8.03, *p* = 5.03E‐16, mm^3^ = 9,032) dorsal anterior cingulate cortex (dACC). See Table 3, for a full overview of the clusters and statistics and Figure 4.

We first verified regions that were systematically activated during a 2‐back working memory task or a cognitive reappraisal task compared to a control task (i.e., 2‐back > control and reappraisal > control).

**FIGURE 2:**
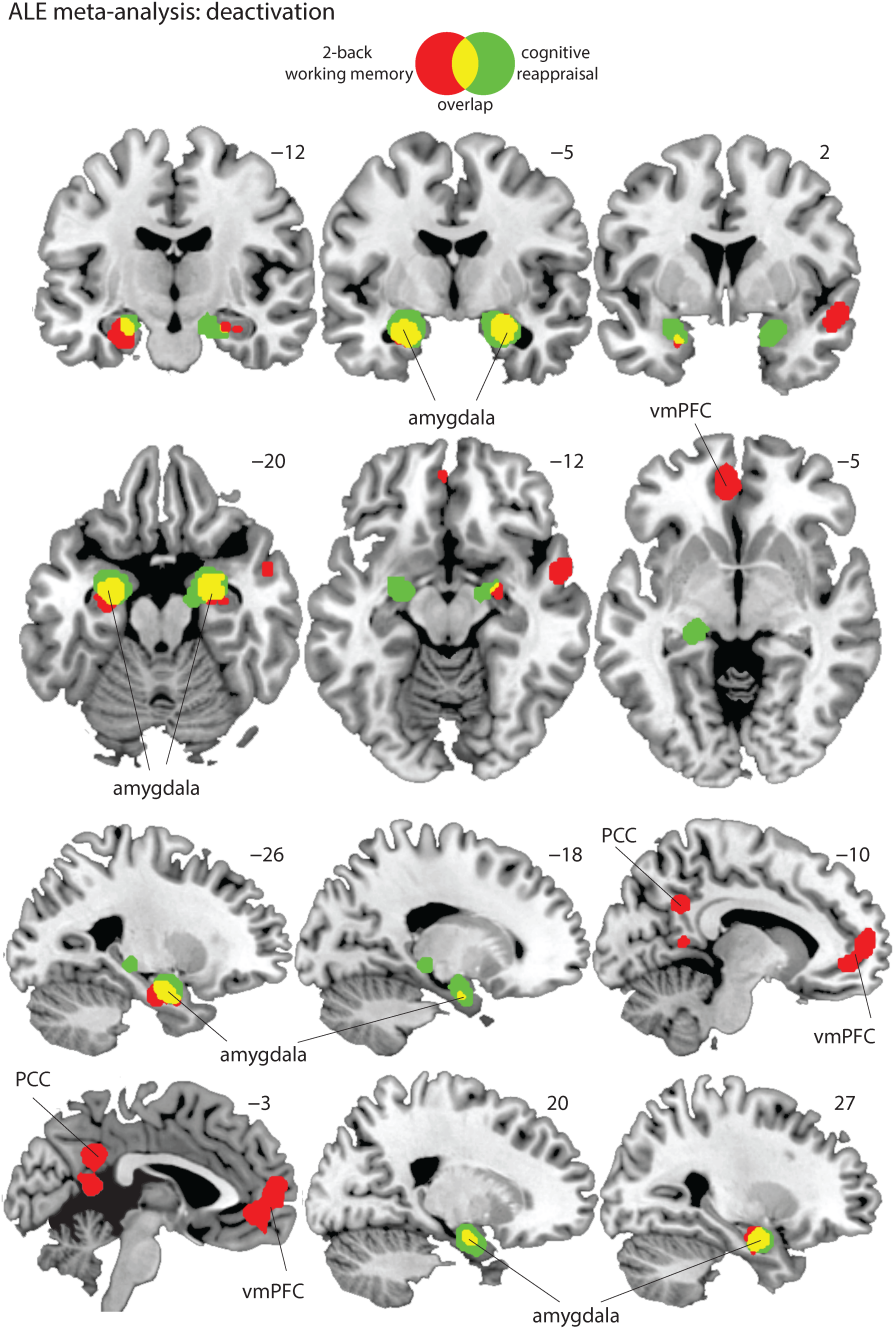
Display of the significant clusters for the activation likelihood estimation (ALE) meta‐analysis on the activation contrasts 2‐back > control (red) and cognitive reappraisal > control (green) and the overlap (yellow). PCC, posterior cingulate cortex; vmPFC, ventral medial prefrontal cortex

We found nine clusters for the reappraisal > control contrast among which are the left (cluster #3, *z* = 6.91, *p* = 2.46E‐12, and mm^3^ = 7,320) and right (cluster #8, *z* = 5.61, *p* = 1.01E‐08, and mm^3^ = 2,712) dlPFC; the left (cluster #2, *z* = 8.10, *p* = 2.81E‐16, and mm^3^ = 9,136) and right (cluster #4, *z* = 8.44, *p* = 6.53E‐18, and mm^3^ = 18,840 and cluster #6, *z* = 7.69, *p* = 7.60E‐15, and mm^3^ = 6,552) inferior frontal gyrus/anterior insula; and the left/right (cluster #1, *z* = 8.80, *p* = 6.66E‐19, and mm^3^ = 10,880) dACC. See Table 3, for a full overview of the clusters and statistics and Figure 2.

Together these findings are in line with previous meta‐analyses' reports of activation patterns during working memory (Wager & Smith, 2003), a 2‐back working memory task (Lee & Xue, 2018), and a cognitive reappraisal task (Buhle et al., 2014; Kohn et al., 2014; Lee & Xue, 2018).

### 3.2 ALE meta‐analysis deactivation contrasts

The main aim of this study was to investigate whether the amygdala is systematically downregulated during working memory in a similar fashion as it is during emotion regulation.

Indeed, for the control > 2‐back working memory contrast, we saw clusters in the left (cluster #3, *z* = 5.53, *p* = 1.56E‐08, and mm^3^ = 1,952) and right (cluster #4, *z* = 5.70, *p* = 6.16E‐09, and mm^3^ = 1,160) amygdala. These clusters fall for 82.6% within the left amygdala and 91.5% within the right amygdala. We also observed a cluster in left/right (cluster #2, *z* = 6.18, *p* = 3.27E‐10, and mm^3^ = 5,480) ventral medial prefrontal cortex (vmPFC) and the left/right (cluster #1, *z* = 6.63, *p* = 1.68E‐11, and mm^3^ = 5,568) posterior cingulate cortex (see Figure 2 and Table 4).

For the control > reappraisal contrast, we also observed clusters in the left (cluster #2, *z* = 9.02, *p* = 9.55E‐20, and mm^3^ = 2,992) and right (cluster #1, *z* = 7.45, *p* = 4.70E‐14, and mm^3^ = 3,728) amygdala, (cluster #3, *z* = 5.75, *p* = 4.55E‐09, and mm^3^ = 952) which overlap with the amygdala clusters found during the control > 2‐back contrast. These clusters fall for 75.8% within the left amygdala and 59.2% within the right amygdala (see Figure 2 and Table 4).

**FIGURE 4:**
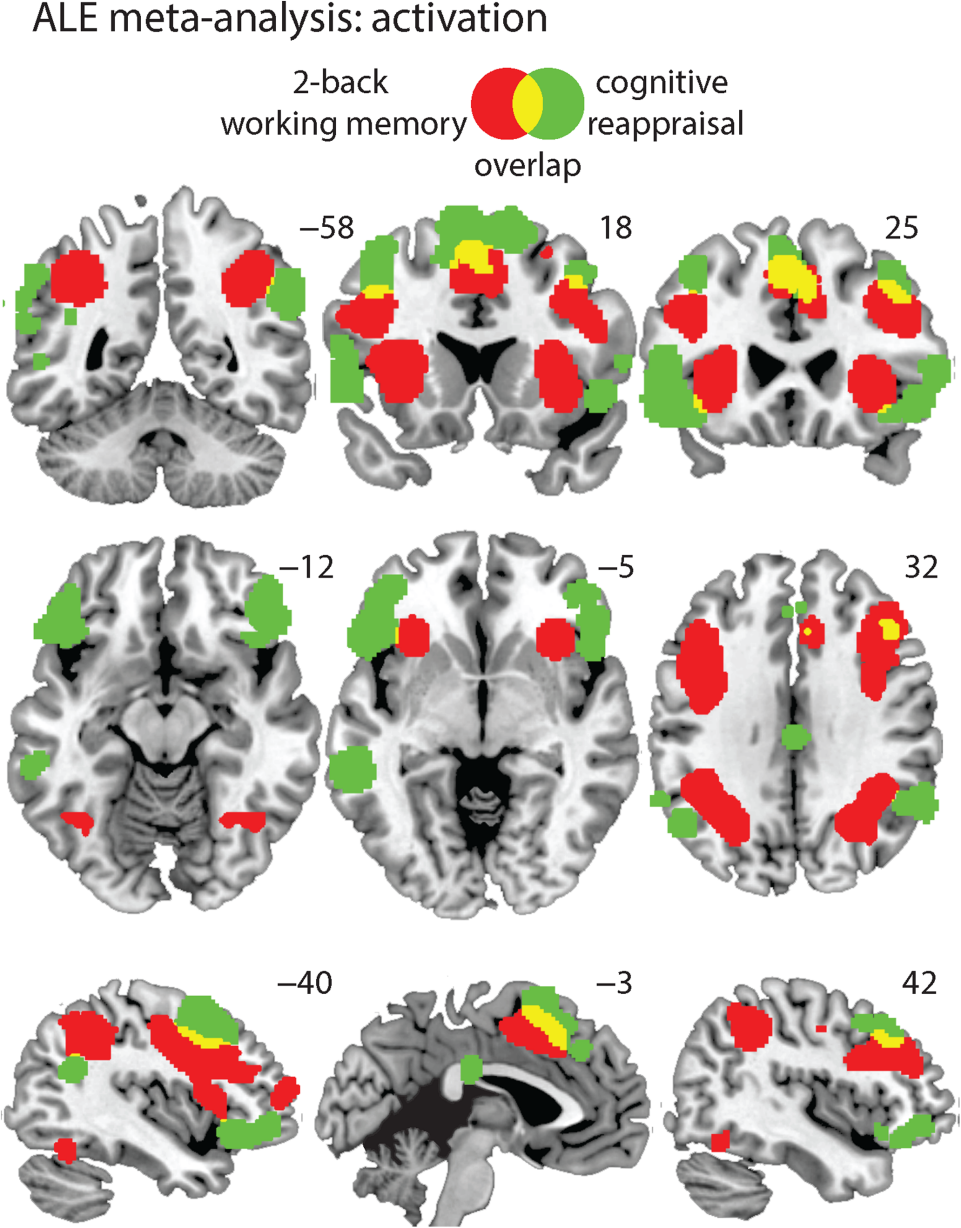
Display of the significant clusters for the activation likelihood estimation (ALE) meta‐analysis. The map from the conjunction analysis showing the similarity in clusters between the “control > 2‐back” and “control > cognitive reappraisal” contrast maps is shown in yellow. In red and green, the result of the subtraction analysis in which the “control > 2‐back” (red) and “control > cognitive reappraisal” (green) contrast maps are directly subtracted from each other is shown, thereby showing distinct regions involved in either of the two tasks

In sum, there is reduced amygdala activity during cognitive reappraisal compared to a control task, as has been shown before (Buhle et al., 2014). Critically, this is also the case during a 2‐back working memory task compared to a control task.

### 3.3 Comparison analysis of the deactivation contrasts

Finally, we performed two comparison analyses between the deactivation contrasts (control > 2‐back and control > reappraise). The first conjunction analysis, aimed at indicating overlapping regions between working memory and cognitive reappraisal, revealed that there is an overlap in deactivation patterns in the amygdala (left: 96.7% falls within the amygdala, right: 91.1% falls within the amygdala) during cognitive reappraisal and the 2‐back working memory task.

The second subtraction analysis was aimed at indicating regions that are distinctly downregulated during working memory or cognitive reappraisal. This analysis revealed that a cluster partly falling within the amygdala (left: 55% falls within the amygdala, 30% falls in the dorsal entorhinal cortex (BA34)) was present stronger for cognitive reappraisal than the 2‐back working memory task, and a cluster partly falling within the amygdala (left: 5% falls in the amygdala, 90% falls in the hippocampus) was present for the 2‐back working memory task compared to cognitive reappraisal.

**FIGURE 3:**
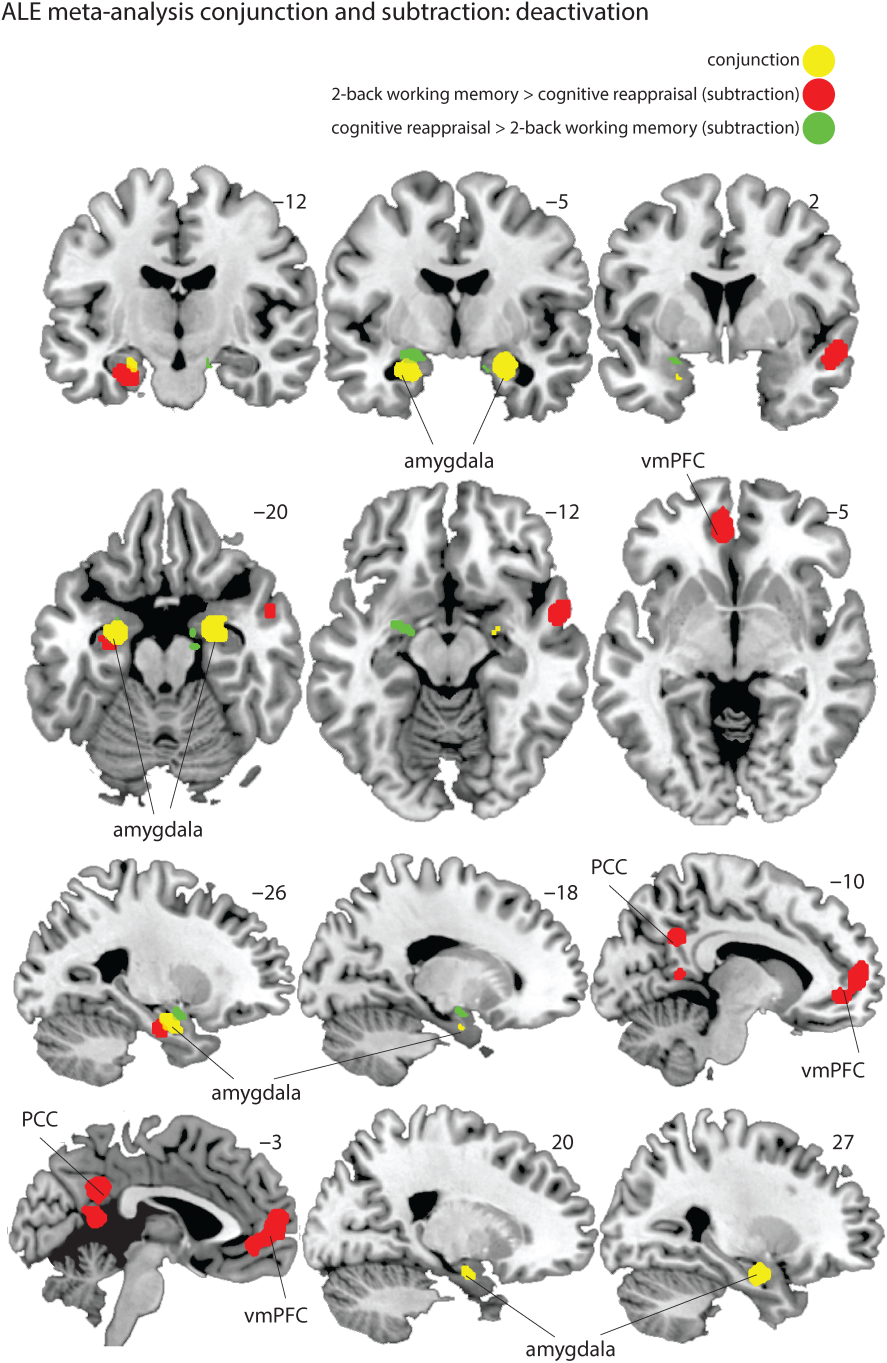
Display of the significant clusters for the activation likelihood estimation (ALE) meta‐analysis on the deactivation contrasts control > 2‐back (red) and control > cognitive reappraisal (green) and the overlap (yellow). PCC, posterior cingulate cortex; vmPFC, ventral medial prefrontal cortex

In sum, although the deactivation clusters associated with both tasks do differ somewhat in their topography, both 2‐back working memory and cognitive reappraisal tasks show bilateral clusters of common deactivations in the amygdala (see Figures 3 and Table 5).

**TABLE 5:** Significant clusters from the ALE meta‐analysis comparing the deactivation patterns

Cluster #	Region	Side	*X* (mm)	*Y* (mm)	*Z* (mm)	mm^3^	ALE	*p*	Peak *Z*
Conjunction									
#1	Amygdala	L	−24	−8	−22	1,232	0.023	NA	NA
#2	Amygdala	R	24	−6	−20	1,064	0.024	NA	NA
									
2‐back > reappraisal									
#1	Posterior cingulate cortex/precuneus	L/R	−1	−51	29	5,048	NA	<0.001	3.89
			−6	−49	16		NA	1.00E‐04	3.72
			−4	−56	18		NA	8.00E‐04	3.16
			14	−56	28		NA	0.001	3.09
#2	Ventromedial prefrontal cortex (vmPFC)	L/R	−2	59	8	5,008	NA	<0.001	3.89
#3	Angular gyrus	L	−49	−66	30	1,120	NA	1.00E‐04	3.72
			−49	−62	23		NA	0.001	3.04
#4	Middle temporal gyrus	R	53	3	−18	872	NA	1.00E‐04	3.72
			58	7	−14		NA	3.00E‐04	3.43
#5	Amygdala/hippocampus	L	−30	−12	−24	560	NA	0.006	2.51
#6	Precuneus	R	8	−58	22	32	NA	0.019	2.07
Reappraisal > 2‐back									
#1	Amygdala/dorsal entorhinal cortex (BA34)	L	−24	0	−14	616	NA	0.004	2.64
#2	Dorsal entorhinal cortex (BA34)	R	14	−6	−20	40	NA	0.035	1.81

*Note*: All coordinates are defined in MNI152 space. All statistics listed are significant at *p* < .05.

Abbreviation: ALE, activation likelihood estimation.

### 3.4 ALE meta‐analysis of increase and decrease reappraisal conditions

To investigate whether the instruction to increase one's emotion using cognitive reappraisal elicits similar activations in the executive control network but increases amygdala activation (instead of the deactivation we observed during a decrease condition), we performed another (control) meta‐analysis on 10 studies that also included an increase condition. This is a low number of studies and the results should therefore be considered with caution.

**FIGURE 5:**
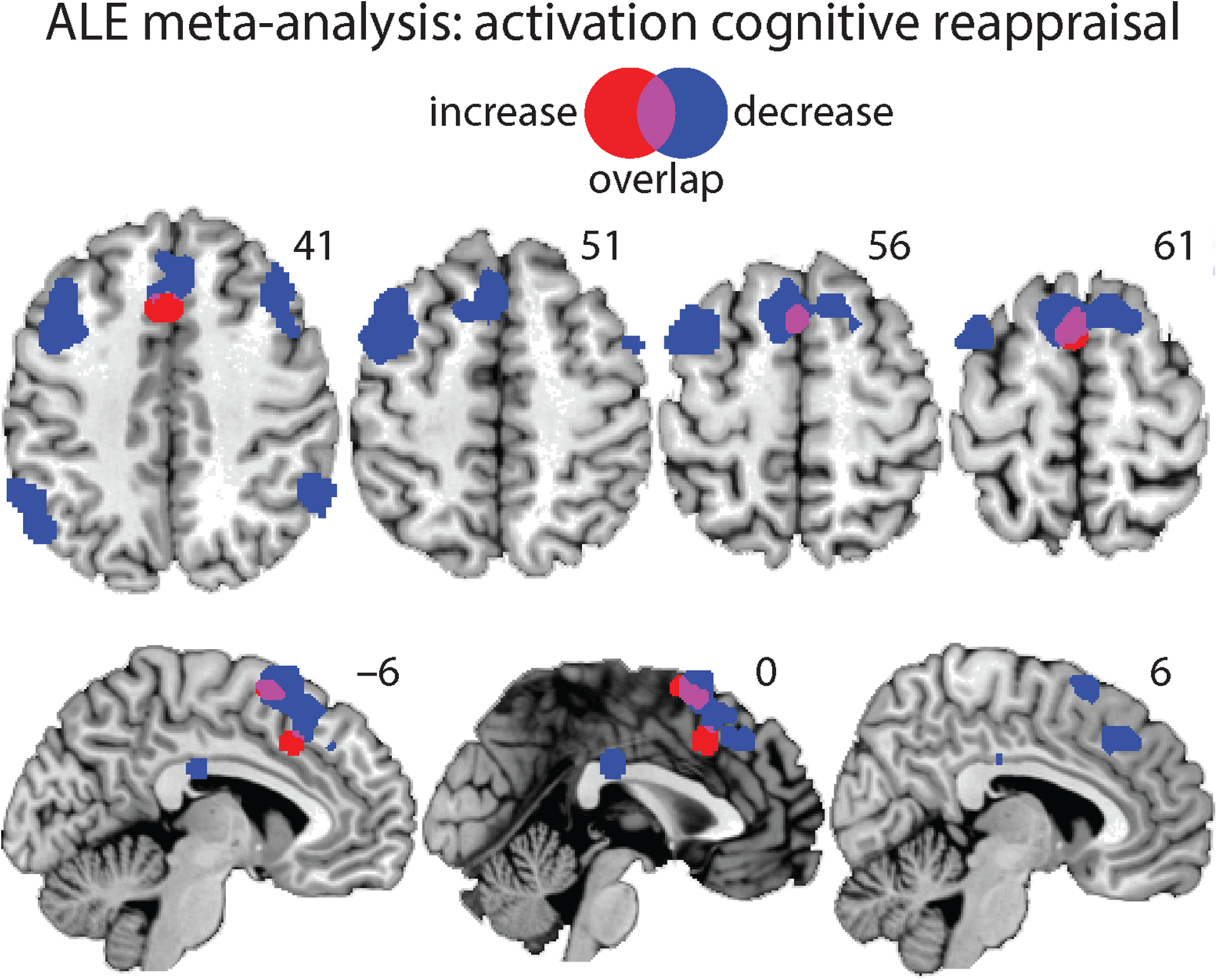
Display of the significant clusters for the activation likelihood estimation (ALE) meta‐analysis on the activation contrasts increase reappraisal > control (red) and decrease reappraisal > control (blue) and the overlap (purple)

We found two clusters for the reappraisal increase > control contrast that are located in the dACC (cluster #1, *z* = 4.64, *p* = 1.727E‐6, and mm^3^ = 1,184; cluster #2, *z* = 5.94, *p* = 1.436E‐9, and mm^3^ = 1,072). The deactivation contrast (control > reappraisal increase) did not reveal any significant clusters (see Figure 5 and Table 6).

Thus, we did not observe consistent activation in the executive control network including the dlPFC nor did we observe a modulation of the amygdala in either direction when participants are instructed to increase their emotions using cognitive reappraisal.

**TABLE 6:** Significant clusters from the ALE meta‐analysis showing activation patterns during the Reappraisal increase condition

Cluster #	Region	Side	*X* (mm)	*Y* (mm)	*Z* (mm)	mm^3^	ALE	*p*	Peak *Z*
Reappraisal increase > control									
#1	Dorsal anterior cingulate cortex	L/R	−2	6	62	1,184	0.018	1.7E‐06	4.64
#2	Dorsal anterior cingulate cortex	L/R	−2	18	40	1,072	0.026	1.4E‐09	5.94

Abbreviation: ALE, activation likelihood estimation.

## 4 DISCUSSION

Using a meta‐analytic approach, we investigated whether a standard working memory task would downregulate the amygdala similar to a cognitive reappraisal task. Reduced amygdala activation is widely considered as a key neural correlate of cognitive regulation of emotion. It has been documented previously in a meta‐analysis of cognitive reappraisal studies (Buhle et al., 2014). We indeed replicate these findings but critically reveal that a working memory task also robustly triggers deactivation in bilateral clusters in the amygdala. This finding also shows that amygdala inhibition can occur without initial amygdala activation in response to acute threat and without an explicit emotion regulation instruction. Together, our findings suggest that amygdala inhibition is likely driven by cognitive demand rather than the content of the cognition.

Downregulation of the amygdala during cognitive reappraisal has typically been interpreted as a top‐down inhibition by prefrontal regions (e.g., Etkin, Egner, & Kalisch, 2011). The amygdala is a region critically implicated in threat detection, as has been detailed in animal models (LeDoux, 1996). Indeed, functional MRI studies in humans have revealed activation of the amygdala related to processing of threatening or salient stimuli (Hariri, Tessitore, Mattay, Fera, & Weinberger, 2002; Morris, Friston, & Dolan, 1997; Vuilleumier, Armony, Driver, & Dolan, 2001). Via reinterpretation of the threatening situation, with the explicit goal to change the affective impact of the threat, such amygdala reactivity is thought to be reduced. Amygdala downregulation during cognitive reappraisal was furthermore shown to be enhanced by real‐time fMRI neurofeedback based on dlPFC responsivity (Sarkheil et al., 2015). Since there are little or no direct connections between the dlPFC and the amygdala (Amaral et al., 1992), downregulation is thought to occur indirectly via the ventromedial prefrontal cortex (e.g., Diekhof et al., 2011; Etkin et al., 2015; Phelps et al., 2004; Schiller & Delgado, 2010), a region involved in implicit forms of emotion regulation such as extinction learning (Hartley & Phelps, 2010). Thus, the commonly held view is that the act of cognitive reappraisal, through neural pathways that are shared with other emotion regulation strategies, leads to a downregulation of the amygdala reactivity to threat.

However, our findings demonstrate that a standard working memory task is also accompanied by a downregulation of the amygdala. This suggests that the content of the cognitive task may not be relevant. While at odds with theories of cognitive reappraisal, this notion is in line with theories postulating a reciprocal relationship between large‐scale neural systems encompassing dlPFC (the executive control network) and amygdala (Drevets & Raichle, 1998; Hermans, Henckens, Joëls, & Fernández, 2014). For instance, acute threat is known to trigger activation of the salience network, and this is accompanied by a loss of executive control network function (Hermans et al., 2014). Most evidence for this comes from studies that have investigated the impact of acute threat and arousal on executive functioning. For example, behavioral studies have shown that during high states of arousal, working memory performance is impaired (Elzinga & Roelofs, 2005; Lupien, Gillin, & Hauger, 1999). This trade‐off also occurs at the network level, namely when participants perform a working memory task while under threat, BOLD signal in the executive control network is reduced compared to a nonthreatening context (Van Ast et al., 2016). Furthermore, the dynamics between the salience network and the central executive control network was shown to change during acute threat (Young et al., 2017).

Our findings suggest that such a trade‐off between the salience network and the executive control network may also occur the other way around. This idea is in line with previous studies indicating that defensive responses, which have shown to be (partly) dependent on the amygdala (Bechara et al., 1995; Klumpers et al., 2015; LaBar et al., 1995), are reduced during cognitively demanding tasks. For instance, during working memory maintenance, threat conditioning is impaired (Carter et al., 2003), and threat‐potentiated startle responses are decreased (Vytal et al., 2012). Other types of cognitively demanding tasks, apart from the 2‐back working memory task we investigated here, also downregulate the amygdala. Examples are playing a game of Tetris (Price et al., 2013) or making goal‐directed eye movements (de Voogd, Kanen, et al., 2018; Jamadar, Fielding, & Egan, 2013). Cognitive demand may indeed lead to a competition between the executive control network and the salience network, where resources are allocated to the executive control network at the expense of the salience network (de Voogd, Hermans, & Phelps, 2018). Thus, the reduced BOLD signal found in the amygdala during cognitive reappraisal and working memory tasks is in line with a vast body of literature showing reciprocal relationships between large‐scale neural systems.

If the executive control network and the salience network are reciprocally activated with respect to one another in both directions, an important question that remains to be answered is how this competitive allocation of resources is established. A first possibility is that resource allocation is established via active suppression. This may occur during a working memory task in a similar fashion as has been proposed for cognitive reappraisal. Namely, downregulation of the amygdala may occur indirectly via the vmPFC (e.g., Diekhof et al., 2011; Etkin et al., 2015; Phelps et al., 2004; Schiller & Delgado, 2010). This mechanism is similar to the proposed working mechanism of implicit emotion regulation such as extinction learning (Hartley & Phelps, 2010), since during extinction, it has been shown the amygdala is inhibited by the vmPFC, leading to a reduction in the expression of threat responses (Milad & Quirk, 2012). Indeed, it has been proposed that the vmPFC may serve as a common mechanism for reducing learned defensive responses to threat (Schiller & Delgado, 2010). This pathway may be activated via several pathways including those involved in high‐order cognition such as the dlPFC, and our findings suggest that the specific content of the cognitive process may not be a critical factor.

It is worthwhile to also consider other potential explanations for the reciprocal relationship between dlPFC and amygdala as observed using functional MRI. One alternative possibility is that when one large‐scale network activates, an increase in blood flow to those regions may deplete other neural systems from oxygenated blood, resulting in decreased BOLD‐fMRI signal. Recent findings indicate that BOLD signal in specific functional brain networks may indeed be partly driven by vascular regulation (Bright, Whittaker, Driver, & Murphy, 2020). The fact that alterations in amygdala‐dependent functions are seen during cognitively demanding tasks that elicit reduced BOLD in the amygdala (Carter et al., 2003; de Voogd, Hermans, & Phelps, 2018; de Voogd, Kanen, et al., 2018; Fox, Zhang, Snyder, & Raichle, 2009; Hermans et al., 2014) appears to speak against the notion that this BOLD signal decrease is a purely vascular effect. However, it is also possible that depletion of oxygenated blood may itself affect neuronal activity. There is indeed evidence that vascular changes can influence neuronal activity (Croal et al., 2015; Hall et al., 2011). Future studies should therefore determine whether amygdala downregulation during cognitively demanding tasks is also observed using electrophysiological methods, which more directly measure neuronal activity.

The topography of the deactivated regions during the 2‐back working memory task and cognitive reappraisal differed slightly. Namely, during the 2‐back working memory task, in addition to the amygdala, we also observed deactivation patterns within the hippocampus and vmPFC. These regions are typically considered part of the default‐mode network (Raichle et al., 2001). It is possible that a 2‐back working memory task and cognitive reappraisal induce qualitatively different deactivation patterns. However, our interpretation is that these differences are more likely due to a difference in cognitive demand between the tasks. Indeed, the magnitude of the deactivation patterns is found to increase with increasing cognitive demand (de Voogd, Hermans, & Phelps, 2018). It is important to note that the overlap in the amygdala does not per definition mean the overlap in amygdala deactivation patterns is due to the same underlying mechanism. Therefore, to establish whether cognitive reappraisal can induce load‐dependent deactivation, future studies should incorporate such a load manipulation within the experimental design. Nevertheless, our findings taken together with previous evidence suggest that cognitive demand, beyond mere attention reorientation or distraction, may play a role in the downregulation of the amygdala that is observed during emotion regulation.

As a control analysis, we have also performed a meta‐analysis on the cognitive reappraisal condition in which participants are instructed to increase their emotions. This analysis was performed on 10 studies only and the results should therefore be considered with caution. Individual studies have indicated that such an “increase” condition activates the executive control network similar to a “decrease” condition (e.g., Domes et al., 2010; Ochsner et al., 2004), while in contrast with “decrease” conditions, amygdala activation is increased (e.g., Ochsner et al., 2004). If this would indeed be the case, it would contradict our theoretical account that the effects of cognitive reappraisal on the amygdala may be driven by cognitive demand rather than the content of the reappraisal. However, not all studies reporting “increase” conditions have found executive control network or dlPFC activation (e.g., Korb, Frühholz, & Grandjean, 2014; Leiberg et al., 2012). In agreement, we did not find evidence for this effect in our meta‐analysis. We also did not find meta‐analytic evidence for consistent activation of the amygdala in “increase” conditions. This outcome appears to be in line with behavioral data showing that increasing one's emotion is not as subjectively effortful as decreasing one's emotion (Ochsner et al., 2004). It is possible that increasing one's emotion increases attention and vigilance toward the emotional information rather than cognitively controlling the emotional response. For example, emotional images that were accompanied by the instruction to increase one's emotions are better recalled a week later than when they are accompanied by the instruction to decrease one's emotions (Ahn et al., 2015). Moreover, van Reekum et al. (2007) showed that during the increase condition participants fixate on the emotional parts of emotional images while they tend to look away during the decrease condition. Together, these data indicate that increasing one's emotions may not be similar to decreasing such responses with regard to the cognitively demanding nature of the task. In line with the outcome of the meta‐analysis, it is therefore not expected that they involve similar neural pathways.

If a cognitively demanding task can reduce threat‐related processes (Carter et al., 2003; Vytal et al., 2012) via downregulation of the amygdala, this may have clinical implications. Indeed, laboratory studies have shown that making cognitively demanding eye movements (de Voogd, Kanen, et al., 2018) or a working memory task (de Voogd & Phelps, 2020; Loos et al., 2020) embedded during extinction learning reduces defensive responses to threat in healthy (de Voogd, Kanen, et al., 2018; de Voogd & Phelps, 2020) and phobic (Loos et al., 2020) participants. These cognitively demanding tasks during extinction learning were accompanied by downregulation of the amygdala (de Voogd, Kanen, et al., 2018; Loos et al., 2020). It could therefore be the case that an additional inhibition of the amygdala during extinction can strengthen safety learning.

If indeed cognitive demand is the mechanism underlying cognitive reappraisal, then any task that is cognitively demanding may potentially be a suitable intervention to reduce defensive responses to threat and potentially have added value in a clinical setting. An ideal intervention, however, should allow for the cognitive demand to be systematically increased to accommodate individual differences in cognitive capacity. The cognitive demand of a working memory task can be systematically increased and has a greater impact on the reduction of BOLD signal in the amygdala (de Voogd, Hermans, & Phelps, 2018). In comparison with cognitive reappraisal, which is one of the most common cognitive emotion regulation strategies translated to the clinic (Kredlow, de Voogd, & Phelps, 2022), compliance with task instructions and task performance in working memory tasks are easier to assess. Since our findings indicate that they operate via similar neural pathways, working memory tasks may have benefits over cognitive reappraisal as a treatment intervention.

It has been argued that distraction during exposure may be counterproductive as it leads to avoidance. It may therefore be the case that performing a cognitively demanding task during treatment may induce distraction and thereby avoidance. However, empirical evidence suggests that in some cases, distraction may be more beneficial than focused exposure (see, for a review, Podinǎ, Koster, Philippot, Dethier, & David, 2013). Moreover, goal‐directed eye movements as used in EMDR could also be seen as distraction but have been shown to have beneficial effects on threat‐related symptoms compared to exposure or extinction alone (de Voogd, Kanen, et al., 2018; de Voogd & Phelps, 2020; Lee & Cuijpers, 2013).

There are a few limitations that are worth mentioning. We observed that only a subset of the articles included in our meta‐analysis reported a deactivation contrast. This was the case for the 2‐back working memory studies (i.e., 16 of the 66 studies) and the cognitive reappraisal studies (i.e., 29 of the 65 studies). It is possible that underreporting of deactivation contrasts has consequences for the conclusion of our findings. We cannot rule out that a systematic bias has led to the decision to report or not to report deactivation patterns. It may be that studies that have reported deactivation patterns may have done so because the results were in line with the expectation. This may be specifically true for cognitive reappraisal studies, as amygdala downregulation forms an important part of the mechanistic explanation of how reappraisal is established. Moreover, we observed that from the studies that contributed to the amygdala deactivation during cognitive reappraisal, 12 of the 16 reported amygdala deactivations based on small volume correction (SVC), while only one of the six studies that contributed to the amygdala deactivation during working memory reported amygdala deactivation based on SVC. It is therefore possible that this bias has led to an overrepresentation of amygdala deactivation for cognitive reappraisal and underrepresentation for working memory. As only 16 of the 29 studies contributed to the amygdala deactivation, it raises the question why some studies report or find amygdala deactivation and others not. This question would be important to address in future research. Nevertheless, the cognitive reappraisal clusters we found overlap with those identified by a large study, and not influenced by a reporting bias, of the Human Connectome study in which 486 participants completed a 2‐back working memory task (The WU‐Minn Human Connectome Project, 2016; Van Essen et al., 2013). We propose that patterns of downregulation are meaningful and that it is therefore important to report BOLD deactivation patterns as well. This will ultimately contribute to a broader understanding of the role of network dynamics in the brain and its relation to function.

In addition, if cognitive demand is indeed driving amygdala downregulation it does not mean that it also is driving changes in self‐report. It is possible that changes in self‐report may occur via other neural pathways also shown to be involved in regulating emotions (e.g., Etkin et al., 2015), apart from potential demand characteristics. This could be a potential explanation why amygdala downregulation and changes in self‐report do not always co‐occur. Future studies could focus on a potential causal relationship between the amygdala and changes in self‐report during cognitive reappraisal, for example, using novel neuromodulatory techniques such as transcranial focused ultrasound stimulation that are currently emerging (e.g., Folloni et al., 2019; Kim et al., 2021).

Although we observed a striking overlap in amygdala deactivation between working memory and cognitive reappraisal, we also observed that the overlap was not absolute. We observed two deactivation clusters in the left amygdala that were unique for either cognitive reappraisal or working memory. For cognitive reappraisal, this deactivation was located dorsally with respect to the conjunction deactivation, within the amygdala and Brodmann area 34. For working memory, the location of the deactivation was more ventral, within the amygdala and hippocampus. This can be interpreted in a few ways. First, it is possible that the deactivation across the two tasks is not identical and both lead to a deactivation pattern that is unique to the task that is being conducted. Second, the difference in topology could be a methodological consequence (e.g., spatial smoothing). Third, an alternative explanation could be that the difference is due to a bias in reporting. Since the amygdala deactivation during cognitive reappraisal is largely based on an SVC, it is possible that this influences the location of the reported peak voxel (i.e., this would always lie within the amygdala). Several studies have shown that deactivation patterns during a working memory task are present in both amygdala and hippocampus (Cousijn et al., 2010; de Voogd, Kanen, et al., 2018; Qin, Hermans, van Marle, Luo, & Fernández, 2009). It is therefore possible that with an SVC, the reporting of the peak value is more biased toward the hippocampus in working memory studies. To resolve this, a study directly comparing working memory and cognitive reappraisal would be necessary to investigate whether the deactivation patterns are similar or meaningfully distinct.

In conclusion, using meta‐analytic evidence, we demonstrate that both cognitive reappraisal tasks and working memory tasks deactivate the amygdala, thus suggesting that the amygdala deactivation is driven by cognitive demand rather than the actual reinterpretation of a threatening stimulus. Our findings are in line with accounts of brain function in terms of reciprocal activation or competition between large‐scale neural networks.

## CONFLICT OF INTERESTS

The authors declare no conflict of interest.

## Data Availability

Data are available on Open Science Framework: https://osf.io/3gqez/?view_only=992a6622e7d94211a69366c515ffdff8.
